# *Brucella abortus* Strain 2308 Wisconsin Genome: Importance of the Definition of Reference Strains

**DOI:** 10.3389/fmicb.2016.01557

**Published:** 2016-09-29

**Authors:** Marcela Suárez-Esquivel, Nazareth Ruiz-Villalobos, Amanda Castillo-Zeledón, César Jiménez-Rojas, R. Martin Roop II, Diego J. Comerci, Elías Barquero-Calvo, Carlos Chacón-Díaz, Clayton C. Caswell, Kate S. Baker, Esteban Chaves-Olarte, Nicholas R. Thomson, Edgardo Moreno, Jean J. Letesson, Xavier De Bolle, Caterina Guzmán-Verri

**Affiliations:** ^1^Programa de Investigación en Enfermedades Tropicales, Escuela de Medicina Veterinaria, Universidad Nacional de Costa RicaHeredia, Costa Rica; ^2^Department of Microbiology and Immunology, Brody School of Medicine, East Carolina UniversityGreenville, NC, USA; ^3^Instituto de Investigaciones Biotecnológicas “Dr. Rodolfo A. Ugalde”, Instituto Tecnológico de Chascomús, Universidad Nacional de San Martín, Consejo Nacional de Investigaciones Científicas y Técnicas, Comisión Nacional de Energía Atómica, Grupo Pecuario, Centro Atómico EzeizaBuenos Aires, Argentina; ^4^Centro de Investigación en Enfermedades Tropicales, Facultad de Microbiología, Universidad de Costa RicaSan José, Costa Rica; ^5^Center for Molecular Medicine and Infectious Diseases, Department of Biomedical Sciences and Pathobiology, Virginia–Maryland College of Veterinary Medicine, Virginia TechBlacksburg, VA, USA; ^6^Wellcome Trust Sanger InstituteHinxton, UK; ^7^Department of Functional and Comparative Genomics, Institute of Integrative Biology, University of LiverpoolLiverpool, UK; ^8^The London School of Hygiene and Tropical MedicineLondon, UK; ^9^Instituto Clodomiro Picado, Universidad de Costa RicaSan José, Costa Rica; ^10^Unité de Recherche en Biologie des Microorganismes, Université de Namur Namur Belgium

**Keywords:** *Brucella*, Brucella abortus, reference genome, reference strain, WGS

## Abstract

Brucellosis is a bacterial infectious disease affecting a wide range of mammals and a neglected zoonosis caused by species of the genetically homogenous genus *Brucella*. As in most studies on bacterial diseases, research in brucellosis is carried out by using reference strains as canonical models to understand the mechanisms underlying host pathogen interactions. We performed whole genome sequencing analysis of the reference strain *B. abortus* 2308 routinely used in our laboratory, including manual curated annotation accessible as an editable version through a link at https://en.wikipedia.org/wiki/Brucella#Genomics. Comparison of this genome with two publically available 2308 genomes showed significant differences, particularly indels related to insertional elements, suggesting variability related to the transposition of these elements within the same strain. Considering the outcome of high resolution genomic techniques in the bacteriology field, the conventional concept of strain definition needs to be revised.

## Introduction

*Brucella* is a bacterial genus responsible for brucellosis, a disease in animals causing infertility, pre-term birth, or abortion (Moreno and Moriyón, 2006). It is also one of the most worldwide spread bacterial zoonosis, not only causing human suffering but also representing a significant economic burden on animal industries. Because this severe and debilitating disease has not been adequately addressed at some national and international level, it is considered by WHO (2014) as one of the “forgotten neglected zoonosis,” constituting a major burden for poor rural communities.

The main etiological agent of cattle brucellosis is *B. abortus* associated with abortion, infertility, reproductive failure, and decreased milk production (Harmon et al., 1989; Xavier et al., 2009; Neta et al., 2010). Humans usually become infected when in contact with infected animals or their derived products, particularly non-pasteurized dairy products (Spink, 1956). Several *B. abortus* reference strains have been described and used as models to understand *Brucella* pathogenesis or as challenge strains for vaccine testing (Meyer and Morgan, 1973; Chain et al., 2005; Halling et al., 2005; Singh et al., 2015).

Genetic drift causing loss of virulence or antigenic properties has been reported in several *Brucella* vaccines strains (Bosseray, 1991; Mukherjee et al., 2005; Jacob et al., 2006; Miranda et al., 2013), but very little is known on the genetic stability of reference strains.

*Brucella abortus* strain 2308 was originally described as a highly virulent strain recovered in 1940 from an aborted fetus of a cow which had been in contact with cattle experimentally infected with a mixture of *B. abortus* cultures (Jones et al., 1965). Since then it has been widely used as a reference and challenge strain within the brucellosis research community (Trant et al., 2010; Dabral et al., 2014; Miranda et al., 2015; Olsen et al., 2015; Truong et al., 2016; Zhu et al., 2016). Whole Genome Sequencing (WGS) of this strain was first carried out by Chain et al. (2005), who found conservation in chromosome synteny with other sequenced *B. abortus* genomes and also some differences that were suggested to be strain specific. In 2009, the results of a WGS analysis from a strain named *B. abortus* 2308A were publically available under accession number GCA_000182625.1. No additional information regarding the isolates used in these two WGS projects was provided.

We performed WGS, functional annotation and manual curation of reference strain *B. abortus* 2308 kept at the Tropical Disease Research Program, Veterinary School, National University in Costa Rica and compared it with these two published *B. abortus* 2308 genomes. Significant differences were found among the genomes, challenging the idea of reference strains as a non-changing entities in time and among laboratories. The use and communication of standardized quality control and experimental design protocols could help interpretation and follow up of reported results.

## Materials and Methods

### Strain Description

All procedures involving live *B. abortus* were carried out according to the “Reglamento de Bioseguridad de la CCSS 39975-0,” year 2012, after the “Decreto Ejecutivo #30965-S,” year 2002 and research protocol approved by SIA 0434-14 from the National University, Costa Rica. A vial of *B. abortus* strain 2308 was obtained at the Tropical Disease Research Program, Veterinary School, National University, Costa Rica, from Dr. Ignacio Moriyón at University of Navarra, Pamplona, Spain. He received it in 1983 from Dr. Lois M. Jones from the laboratory of Prof. David T. Berman at University of Wisconsin Madison as a lyophilized vial coming originally from the National Animal Disease Center at Ames, IA, USA. From this, bacteria were expanded in trypticase soy agar, assayed biochemically to assure properties (Alton et al., 1988) and stored at -70°C in 20% glycerol. For master seed preparation, recommendations for identification, maintenance and to rule out attenuation were followed as suggested (Alton et al., 1988). Therefore, before master seed storage, a passage in mice was performed using 10^5^CFU from an overnight culture in 100 μL PBS for intraperitoneal inoculation. Bacteria were recovered from the spleen after 3 weeks after platting in trypticase soy agar and a single colony expanded in trypticase soy broth overnight (Bosseray, 1991; Grilló et al., 2012). Trypticase soy broth aliquots were prepared with 20% glycerol and stored at -70°C as a master seed. Protocols for experimentation with mice were revised and approved by the Comité Institucional para el Cuido y Uso de los Animales of the Universidad de Costa Rica (CICUA-47-12) and were in agreement with the corresponding law, Ley de Bienestar de los Animales, of Costa Rica (law 7451 on animal welfare). Mice were housed in the animal building of the Veterinary School, Universidad Nacional, Costa Rica. Animals were kept in cages with water and food *ad libitum* under biosafety containment conditions previous to and during the experiment.

Finally, a master seed aliquot was grown in tryptic soy agar for phenotypical and biochemical characterization (Alton et al., 1988), MLVA-16 (Le Flèche et al., 2006), Bruce-ladder PCR (López-Goñi et al., 2011) and infection of Raw macrophages as described (Palacios-Chaves et al., 2011). Aliquots were used only once and discharged.

Due to its origin, this strain is from herein referred as *B. abortus* strain 2308 Wisconsin (*B. abortus* 2308W).

### WGS, Assembly and Annotation

A bacteriological loop sample from a frozen master seed aliquot of *B. abortus* 2308W was inoculated in trypticase soy broth and grown overnight. DNA was extracted using the Promega Wizard Genomic DNA Purification kit, and stored at -70°C until used.

WGS was performed at the Wellcome Trust Sanger Institute on Illumina platforms according to *in house* protocols (Quail et al., 2009, 2012), resulting in 4 396 650 reads, depth of coverage: 128.76 ± 34.84, an error rate of 0.005 and a duplication rate of 0.0017.

The SNPs discovery performed in this study used raw reads with a base quality score of Q33. Sequence variation was called by BCFtools^1^ if the depth of coverage is greater than 5, the variant is present in at least 75% of reads at that position, the variant is present on both strands, and the mapping quality is greater than 30. Each SNP was manually investigated using BamView (Carver et al., 2010).

For WGS assembly and alignment, sequencing reads were *de novo* assembled using Velvet Optimiser (Zerbino and Birney, 2008; Page et al., 2016) and 18 contigs (N50 = 294779) were ordered using Abacas (Assefa et al., 2009) against *B. abortus* 9–941 (accession numbers NC_006932 and NC_006933). To detect mis-assemblies, raw data were mapped back against the 2308W genome assembly using SMALT 0.5.8^2^ which resulted in 99.89% mapping. Two more assembly controls were performed by mapping the 2308W raw reads against 2308 reference genome (NC_007618 and NC_007624) or 9–941 reference genome. This resulted in 98.88 and 98.77% mapping, respectively.

Annotation was automatically transferred from a previously WGS obtained from *B. abortus* 2308 (NC_007618 and NC_007624). In order to facilitate the 2308W annotation review, a BLAST comparison (Altschul et al., 1997) between 2308W chromosomes, *B. abortus* 9–941, *B. melitensis* 16M (accession NC_003317.1 and NC_003318.1) and *B. suis* (NC_004310.3 and NC_004311.2) was performed. Visualizations were done with Artemis and BLAST comparisons with the Artemis Comparison Tool (Rutherford et al., 2000). Each coding region (CDS) was checked manually and curation was performed according to available experimental information and literature search on *B. abortus* 2308. Major findings were summarized in **Supplementary Table S1** and a link to an editable version is available at https://en.wikipedia.org/wiki/Brucella#Genomics.

Sequencing info has been deposited at the European Nucleotide Archive (ENA)^3^ under the accession code ERS568782.

### Genome Comparison among *B. abortus* 2308 Strains

A BLAST comparison using the genome here described, *B. abortus* 2308W (assembly accession ERS568782) and two previously published genomes: *B. abortus* 2308 (Chain et al., 2005; accession number NC_007618 and NC_007624); and *B. abortus* str. 2308A (assembly accession GCA_000182625.1; contigs accession numbers ACOR01000001-ACOR01000009) was performed. Visualizations were done with Artemis and ACT (Rutherford et al., 2000).

As the 2308A genome is reported as contigs, raw sequencing data were downloaded and *de novo* assembly using Velvet Optimizer (Zerbino and Birney, 2008) was performed followed by ordering with Abacas (Assefa et al., 2009), using *B. abortus* 9–941 genome as reference as per the analysis of our strain.

## Results And Discussion

For comparative analysis, we performed WGS analysis of the 2308 strain that has been used in our laboratory and referred here as *B. abortus* 2308W, to distinguish its genome from previously reported ones (2308 and 2308A). The genome consists of two chromosomes, chromosome I is 2.10 kb in size and chromosome II is 1.16 kb. Automated annotation and manual curation were performed and summarized in **Supplementary Table S1**. An editable spreadsheet was also created in order to facilitate updates in the annotation or additional relevant comments (e.g., virulence, function, mutants availability) and is available through a link at https://en.wikipedia.org/wiki/Brucella#Genomics.

**FIGURE 1:**
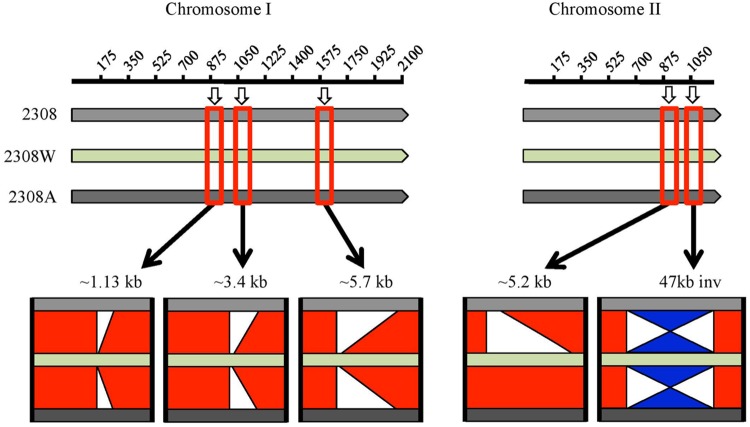
**Graphic representation of BLAST comparison of *Brucella abortus* 2308 (light gray bar), 2308W (light green bar) and 2308A (dark gray bar), visualized in ACT.** Base pairs in kb coordinates are shown in the upper line for each chromosome. The red squares over the bars highlight an approximate region where the main differences among the genomes are found, and a zoom in is represented below. The red bars in the middle of the genomes’ bars indicate the regions are identical. White segments indicate deletions and the blue “hourglass-like” figure means an inversion. Chromosome I of 2308W shows the absence of three regions, which are present in the other two genomes. These regions coincide with contig breaks. At chromosome II, a 5.2 kb insertion is observed in 2308W and 2308A, and an inversion is present in 2308W. All deletions and inversions are surrounded by insertion elements.

A BLAST comparison of this genome with those two previously reported showed major differences summarized in **Figure 1** and detailed in **Supplementary Table S2**. Three major deletions in chromosome I in *B. abortus* 2308W were detected relative to *B. abortus* 2308 and 2308A of 1.13, 3.4, and 5.7 kb. It is important to point out that these three deletions are located at the end of contigs and surrounded by repetitive sequences so mayrepresent incompletely assembled contiguous sequences, rather than genuine deletions. Regions containing repetitive sequences are proven difficult to assemble and sequence, regardless the sequencing technology used (Bidmos and Bayliss, 2014). The 1.13 kb deletion contains the 2308 loci BAB1_0934 to BAB1_0937 associated to IS711 elements and transposases. The 3.4 kb deletion includes loci BAB1_1102–BAB1_1104; the first two CDSs are predicted proteins of unknown function and the third one is predicted to be a site-specific recombinase, DNA invertase. These loci fall within a larger 8.1 kb genomic island named GI-1, encoding mainly predicted proteins and phage-related proteins (Rajashekara et al., 2004). Five CDSs are part of the 5.7 kb region absent in chromosome I of 2308W (BAB1_2221–BAB1_2225). They encode tRNAs and rRNAs genes with copies elsewhere in the genome (**Supplementary Table S2**). These copies are represented by a higher number of reads as compared with average for the rest of the CDS (9700 versus 3200 reads), suggesting that contig breaks are caused by mis-assembly of the repeated copies of the tRNA and rRNA genes during the assembly.

An insertion of a 5.2 kb segment in chromosome II was observed in 2308W and 2308A, relative to 2308 (Chain et al., 2005). This region displayed even read coverage as compared to the rest of the 2308W genome. It includes four genes encoding sugar binding proteins, proteins involved in nitrogen metabolism as well as the transcriptional activator FtrB involved in regulation of carbon and amino acid metabolism (BAW_20862–BAW_20865). The adjacent loci (BAW_20861 and BAW_20866) contain sequence partial deletions in 2308 (corresponding to BAB2_0903) or 2308W (corresponding to BAB2_0904). Also evident is an inversion of the 47 kb GI-5 (Rajashekara et al., 2004) in 2308W as compared to 2308 and 2308A that results in deletion of BAB2_1075, encoding an IS3 transposase. Notably, insertion elements were detected alongside all of the indel regions. These observations show not only how unstable the *B. abortus* 2308 genome is but also that chromosomal rearrangements are a source of genetic variability, probably as consequence of IS mediated transposition events (Tsoktouridis et al., 2003; Ocampo-Sosa and García-Lobo, 2008; Mancilla et al., 2012).

According to the National Collection of Cultures (NCTC) in England, reference strains are “stipulated in internationally recognized standard methods as definitive control strains for various microbiological testing procedures^4^.” The fact that *Brucella* is regarded as a genetically homogeneous genus, but significant differences are present in *B. abortus* 2308 stored in different laboratories raises a broader question and challenges the concept of “reference strain.” In the case of *B. abortus* 2308 this is even more relevant, since this strain has been regarded as the canonical challenge organism in vaccine trials where different results about its survival in mice models are reported (Montaraz and Winter, 1986; Ko et al., 2002; Yang et al., 2006). There are probably several reasons for these contrasting results. Some, such as differences due to strain handling can be accounted by using standardized quality control and experimental design protocols. Probably more difficult to control are genome changes. Reference strains are usually isolates obtained from clinical cases used in research labs as infection models, and as such, are subject of genetic modification according to the environment (Hopkins et al., 1981; Turse et al., 2011). A detailed description of the strain used, including a publically available WGS, as well as growth, propagation and maintenance conditions included with reported results could help readers to better assess the reported observations.

## Author Contributions

MS-E performed bioinformatics analysis, manual curation, and prepared tables and figures. NR-V performed molecular typing and manual curation. AC-Z, CJ-R, RR, and DC performed manual curation. CC provided data for manual curation. EB-C and CC-D performed biochemical typing and provided data for manual curation. KB performed bioinformatics analysis and edited the manuscript. EC-O and EM analyzed data. NT performed bioinformatics analysis and manual curation. XD and JL performed manual curation, organized, and analyzed data. CG-V designed the study, analyzed data, performed manual curation, and wrote the manuscript. All authors edited the final version of the manuscript.

## Conflict of Interest Statement

The authors declare that the research was conducted in the absence of any commercial or financial relationships that could be construed as a potential conflict of interest.

## Footnotes

1 https://samtools.github.io/bcftools/bcftools.html

2 http://www.sanger.ac.uk/resources/software/smalt/

3 http://www.ebi.ac.uk/ena/

4 https://www.phe-culturecollections.org.uk/collections/nctc.aspx

## Supplementary Material

TABLE S1 **Primers used for RT-pPCR analysis for the virulence analysis**.

TABLE S2 **Fungicidal concentration of amphotericin B for planktonic cells and biofilm madure of *Cryptococcus neoformans* and *Cryptococcus gattii***.
